# Dietary Polyphenols as Potential Therapeutic Agents in Type 2 Diabetes Management: Advances and Opportunities

**DOI:** 10.1016/j.advnut.2024.100346

**Published:** 2024-11-19

**Authors:** Sisir Kumar Barik, Srabasti Sengupta, Rakesh Arya, Surendra Kumar, Jong Joo Kim, Reetika Chaurasia

**Affiliations:** 1 The Rowett Institute, University of Aberdeen, Aberdeen, United Kingdom; 2 Department of Neurosurgery, University of Florida, Gainesville, Florida, 32608, United States; 3 Department of Biotechnology, Yeungnam University, Gyeongsan, Gyeongbuk, 38541, Korea; 4 Department of Orthopaedic Surgery, The Johns Hopkins University School of Medicine, Baltimore, MD, 21287, United States; 5 Department of Internal Medicine, Section of Infectious Diseases, Yale University School of Medicine, New Haven, CT, 06510, United States

**Keywords:** postprandial hyperglycemia, glucose transporter, AMPK, glucagon-like peptide 1, berry, polyphenol, anthocyanin, DPP-IV, type 2 diabetes

## Abstract

Poor dietary intake or unhealthy lifestyle contributes to various health disorders, including postprandial hyperglycemia, leading to type 2 diabetes mellitus (T2DM). Reduction of postprandial glucose concentrations through diet is a key strategy for preventing and managing T2DM. Thus, it is essential to understand how dietary components affect glycemic regulation. Dietary polyphenols (DPs), such as anthocyanins and other phenolics found in various fruits and vegetables, are often recommended for their potential health benefits, although their systemic effectiveness is subject to ongoing debate. Therefore, this review assesses the current and historical evidence of DPs bioactivities, which regulate crucial metabolic markers to lower postprandial hyperglycemia. Significant bioactivities such as modulation of glucose transporters, activation of AMP kinase, and regulation of incretins are discussed, along with prospects for diet-induced therapeutics to prevent the onset of T2DM.

**Statement of significance**

This review uniquely synthesizes both current and historical data on the metabolic bioactivities of dietary polyphenols, particularly focusing on their mechanisms—such as modulation of glucose transporters, AMP kinase activation, and incretin regulation—which may help lower postprandial hyperglycemia, offering a more integrated understanding of diet-induced strategies for type 2 diabetes mellitus prevention.

## Introduction

Diabetes is one of the major noncommunicable diseases, along with cardiovascular diseases, cancers, and respiratory diseases. These four noncommunicable diseases cause 41 million deaths annually, accounting for 74% of all premature global fatalities. Diabetes alone is responsible for 1.5 million deaths annually and imposes a significant burden on health care systems [1]. Obesity, lifestyle factors, genetic predispositions, and epigenetics are majorly accountable for type 2 diabetes mellitus (T2DM) progression [2]. The International Diabetes Federation reported that 537 million people had diabetes globally in 2021, resulting in $966 billion in health expenditures, with costs projected to exceed $1054 billion by 2045 [[3], [4], [5]]. Among the global diabetic population, 90% experience T2DM, and 374 million people are at increased risk of developing it [5]. Currently, no effective mechanism exists to prevent T2DM, which can limit its progression to an epidemic. Moreover, the increasing morbidity and mortality from T2DM causes major concerns in the global health sector.

**FIGURE 1:**
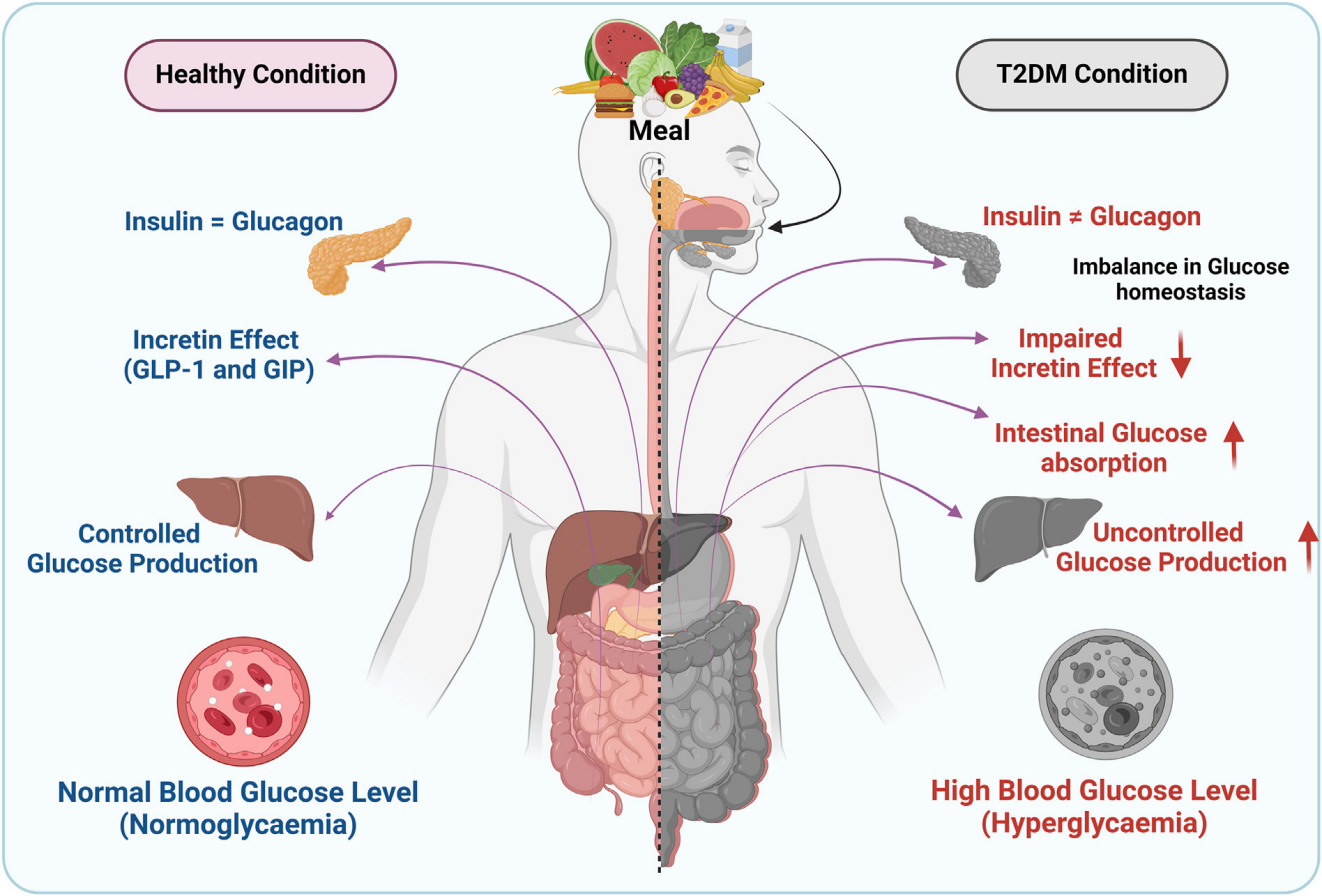
Comparison of a healthy state with the pathophysiology of postprandial hyperglycemia leading to type 2 diabetes mellitus (T2DM). GIP, gastric inhibitory peptide; GLP, glucagon-like peptide.

T2DM primarily arises from persistent postprandial hyperglycemia and hyperinsulinemia, which result in insulin resistance, pancreatic β-cell dysfunction, and inflammation [6]. Insulin resistance impairs proper insulin secretion and signaling, leading to the development of T2DM. In contrast, glucagon stimulates hepatic glycogenolysis and gluconeogenesis during hypoglycemic conditions [7]. Both insulin and glucagon are vital for maintaining fuel homeostasis, being released reciprocally in response to blood glucose fluctuation. During the fed state, insulin dominates by facilitating glucose uptake in target organs, while in fasting conditions, glucagon mobilizes hepatic glucose to maintain normal blood sugar concentrations [7]. In prolonged hyperglycemia, the balance between glucagon and insulin action weakens. During meals, incretin hormones like glucagon-like peptide (GLP) 1 and gastric inhibitory peptide (GIP; also known as glucose-dependent insulinotropic polypeptide) are released from the gut endocrine cells, enhancing glucose-induced insulin release and potentially accounting for ≤70% of postprandial insulin secretion (Figure 1) [8,9]. Impaired incretin action or reduced secretion of incretin hormones may lead to the onset of T2DM, especially in individuals with prediabetic conditions or hyperglycemia [10,11].

Dietary polyphenols (DPs) are naturally occurring compounds found in various plant-based foods such as fruits, vegetables, and whole grains and are known for their antioxidant, anti-inflammatory, and metabolic effects [12,13]. The molecular mechanism of DPs and their natural derivatives have potential hypoglycemic or antidiabetic properties, which could be harnessed pharmaceutically to treat or manage T2DM and help to achieve the Sustainable Development Goal (SDG target 3.4) by reducing premature mortality. This review thus aims to evaluate recent finding and historical data from various reports of DPs, especially anthocyanins from berries, and their impact on glucose transporters, AMP kinase (AMPK) activation, and incretin regulation, which are key targets of diet-induced therapies in the prevention and management of T2DM. The impact of DPs on modulating glucose transporters (GLUTs), AMPK, and incretins are reevaluated, discussed, and hypothesized with their mechanisms of action for future relevance.

## Current Management and Treatment Practices for T2DM: Antidiabetic Drugs and Their Side Effects

There is currently no specific medication that can completely cure T2DM. However, it can be managed with existing drugs and lifestyle changes [14]. Treatments to control hyperglycemia in T2DM include noninsulin hypoglycemic agents such as biguanides (metformin), sulfonylureas, thiazolidinediones (e.g. pioglitazone), and commonly used α-glucosidase inhibitors (e.g. Acarbose) [15]. Metformin, a dimethyl biguanide, is the most used drug for managing increased glycemic levels. It enhances intestinal glucose uptake, elevates the GLP-1 concentrations, and alters the microbiome [11]. However, research indicates that some patients are intolerant to metformin due to reduced organic cation transporter 1 transport in the intestine [16]. The mechanism of action of these antidiabetic drugs and their efficacy in patients experiencing side effects remain poorly understood [17]. Thiazolidinediones are another class of oral antidiabetic drugs that enhance insulin sensitivity by regulating adipocyte differentiation [18]. However, their use can lead to several complications and gastrointestinal side effects, including nausea, diarrhea, stomach pain, vomiting, and loss of appetite, which often occur together [19,20]. Therefore, replacing or supplementing existing antidiabetic drugs with natural compounds having hypoglycemic or improved therapeutic properties to minimal side effects may be beneficial in treating or preventing the development of T2DM.

Studies indicate that T2DM can be prevented or at least managed through a proper diet and healthy lifestyle [21,22]. However, modifiable risk factors for developing T2DM vary across populations and include obesity, overnutrition or undernutrition (including malnutrition in the womb and early life), and physical inactivity [23]. An unhealthy diet leading to excessive body fat is one of the strongest risk factors for developing T2DM. Nutritional transitions that increase risk of T2DM typically involve higher consumption of animal fat, decreased fiber intake, and frequent consumption of fast foods [21]. WHO’s fact sheet on healthy diets for adults recommends consuming ≥400 g (5 portions) of fruits and vegetables daily. These foods contain a wide variety of DPs, which can help in reducing the burden of chronic cardiovascular diseases, obesity, and T2DM [24]. Therefore, an ideal solution to manage the increasing incidence of T2DM is to develop nutritional strategies alongside cost-effective, natural therapeutic/antidiabetic drugs with minimal or no side effects [25]. This approach would benefit not only people with T2DM but also those with prediabetes. Preventing T2DM is more cost-effective than treating its complications, which can be achieved by maintaining a proper diet and healthy lifestyle [26]. On the contrary, the overall health benefits of natural or dietary compounds are uncertain, and caution should be exercised before encouraging their widespread consumption until further research clarifies their effects [27,28].

## Dietary Polyphenols

Phytochemicals with phenol rings are classified as polyphenols, which are further categorized based on their origin, biological function, and chemical structures. DPs are a group of secondary metabolites, that are, organic compounds produced by plants that are not essential for the plant’s normal development but play a vital role in the plant’s defense mechanisms. DPs are commonly found in fruits, vegetables, wine, tea, extra virgin olive oil, chocolate, and other cocoa products. These polyphenols are primarily derivatives and/or isomers of flavones, isoflavones, flavonols, catechins, and various phenolic acids such as caffeic acid, chlorogenic acid, and ferulic acid [12,13]. Flavonoids are the most extensively studied group of DPs, with anthocyanins being particularly notable due to their suggested bioactivities at various cellular targets. Anthocyanins and plant polyphenols have potential antidiabetic properties, which may help regulate postprandial glucose concentrations. They are natural products found in fruits, vegetables, cereals, dry legumes, chocolate, and beverages, such as tea, coffee, or wine [29]. Several studies have shown a link between fruit consumption and reduced postprandial blood glucose concentrations, with soft fruits, especially berries, standing out for their high concentrations of bioactive components, particularly anthocyanins [30,31].

DPs from berries have been used in traditional medicines [32] to treat diabetic symptoms. Recently, they have been recognized as promising candidates for the prevention and management of T2DM due to their various biochemical and cellular bioactivities [33,34]. The bioactive compound glucosylated anthocyanins from highbush blueberries exhibits antioxidant activity and has been found to inhibit carbohydrate-hydrolyzing enzymes such as α-amylase and α-glucosidase. Synthetic drugs such as acarbose, miglitol, and voglibose are commonly used to inhibit the action of α-amylase and α-glucosidase enzymes in patients with T2DM. However, these drugs can cause various side effects, including abdominal distention, flatulence, meteorism, and possibly diarrhea [35].

## Anthocyanins as Insulin Secretagogues

Anthocyanins, which are sugar conjugates of anthocyanidins, are plant metabolites responsible for the pink, red, purple, and blue colors in the epidermal tissues of certain berries, fruits, vegetables, flowers, and grains [36]. The most common dietary anthocyanidins (aglycone form of anthocyanins) are delphinidin, cyanidin, malvidin, pelargonidin, peonidin, and petunidin [37]. Anthocyanins vary depending on number of hydroxyl groups, the attachment site of the sugar units, the type of sugar (e.g. arabinose, galactose, glucose, rhamnose, and xylose frequently encountered), glycosidic linkage (α or β linkage), and complexity of the sugars (monosaccharides, disaccharides, and trisaccharides) [38]. Anthocyanins are proposed as insulin secretagogues, with the first report of insulin secretion induced by these compounds in pancreatic β cells reported by Jayaprakasam et al. [39]. Anthocyanins influence adipocyte function, potentially limiting the development of obesity and related metabolic diseases [40,41].

Preclinical data indicate that anthocyanins regulate adipose tissue metabolism by improving adipocyte dysfunction, enhancing β-oxidation, and reducing fat accumulation on adipocytes [42]. Extractable and nonextractable fractions of table grapes in male C57BL/6J mice demonstrated that the anthocyanin-rich extractable fractions mitigated metabolic consequences such as decreased adiposity, improved insulin resistance, and reduced markers of inflammation associated with a high-fat diet [43].

**FIGURE 2:**
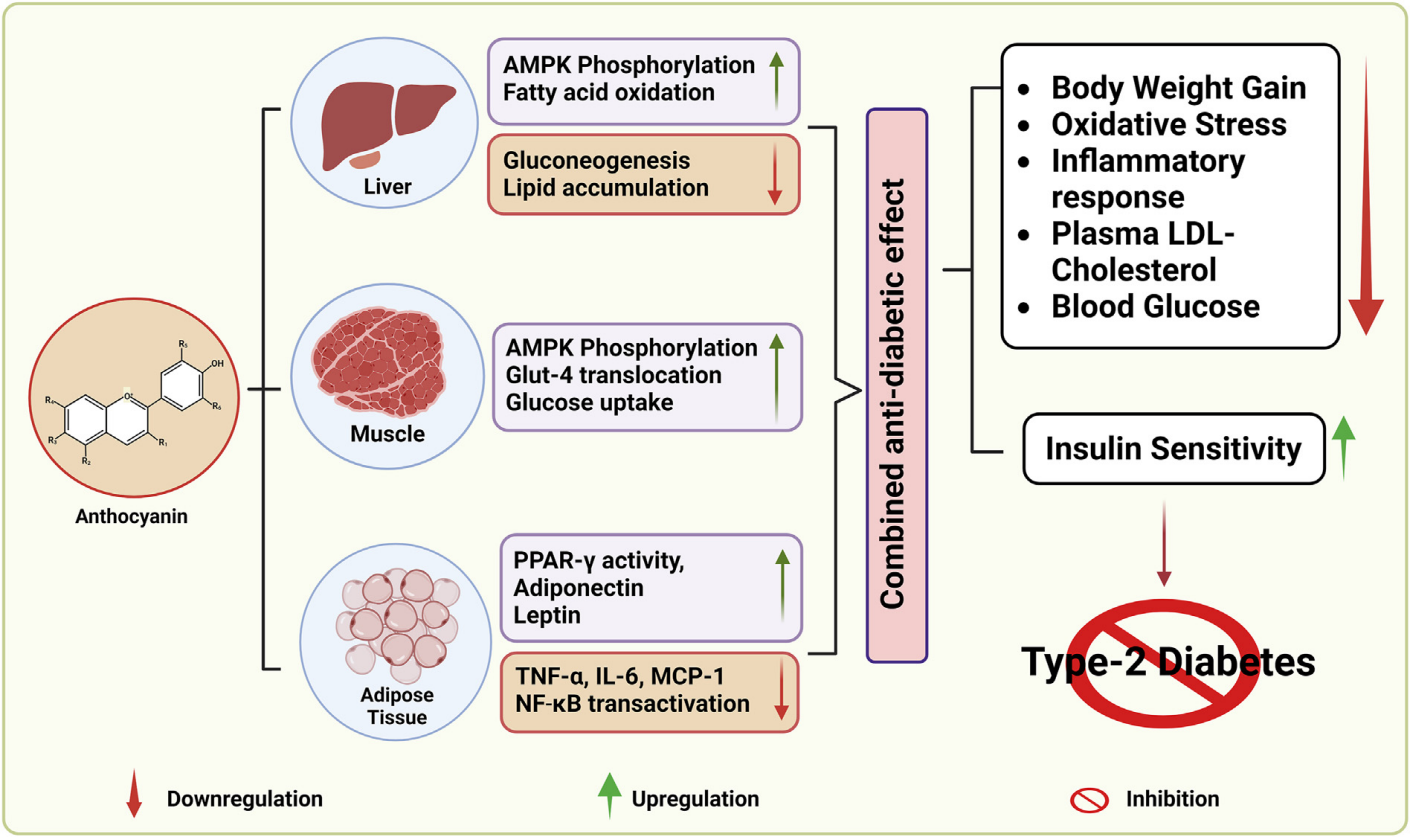
Anthocyanin exerts a combined antidiabetic effect through several mechanisms. They enhance insulin sensitivity while reducing body weight gain, oxidative stress, inflammatory response and plasma LDL-cholesterol and blood glucose concentrations. These effects involve interactions with key factors other than AMP kinase (APMK) phosphorylation or modulating glucose uptake in various organs, such as peroxisome proliferator-activated receptor (PPAR) γ, TNF-α, IL-6, monocyte chemoattractant protein (MCP)-1, and nuclear factor (NF)-κ light-chain enhancer of activated B cells.

Epidemiologic and cohort studies indicate that increased consumption of anthocyanins may reduce risk of developing T2DM, suggesting that overweight or obese individuals should consider consuming more anthocyanin-rich foods to prevent the onset and progression of T2DM [44,45]. The potential mechanisms underlying the antidiabetic properties of anthocyanins may involve inhibiting body weight gain, preventing the production of free radicals and lipid peroxidation, regulating inflammatory response, lowering blood glucose and lipids, and improving insulin resistance (Figure 2) [45]. These studies suggest that anthocyanins interact with various complex cellular signaling pathways, including transcription factors and associated enzymes, synergistically producing hypoglycemic and antidiabetic effects.

The pharmacokinetic profile derived from various in vivo and in vitro studies suggests that anthocyanins are absorbed, metabolized, distributed, and excreted with beneficial health implications [46,47]. According to Matsumoto et al., based on animal [48] and human studies [49], anthocyanins are rapidly absorbed within 6 and 20 min after ingestion and reach the bloodstream intact within 15 and 60 min. However, de Ferrars et al. [50] concluded that their clearance involves multiple processes, including enterohepatic recirculation, hepatic recycling, and microbial metabolism, with prolonged intestinal absorption occurring from both the small and large intestines. Mazza et al. [51] observed that 0.002%–0.003% (or 20–30 ppm) of anthocyanins were detected in human serum 3 h after ingestion, a finding corroborated with the results reported by Matsumoto et al. [49]. The correlation between serum anthocyanin concentrations and enhanced postprandial antioxidant status has been significantly demonstrated with berries consumption, owing to their high anthocyanin contents [52,53]. Therefore, extensive research but limited clinical studies suggest that dietary anthocyanins, particularly those from soft fruits like berries, could potentially improve insulin resistance and provide health benefits in diabetic conditions.

## Impact of DPs on Carbohydrate Digesting Enzymes and Intestinal GLUTs

DPs, especially those from berries, have demonstrated effectiveness in reducing sucrose digestion and absorption, leading to a delayed glycemic response [30,54]. DPs target specific sites, primarily digestive enzymes and cellular glucose uptake in various parts of the gastrointestinal tracts—such as the mouth, stomach, and small and large intestines for diverse health effects. However, despite their immediate contact in the digestive tract postintake, the mechanisms of action of these bioactive compounds in these setting remains elusive [31]. It is conceivable that berry polyphenols act in the digestive tract initially by inhibiting α-amylases in the mouth, subsequently inhibiting the action of α-glucosidases and modulating sugar transporters, such as sodium-dependent glucose cotransporter (SGLT) 1, GLUT2, and GLUT5, across the small intestine to potentially reduce risk of T2DM.

**FIGURE 3:**
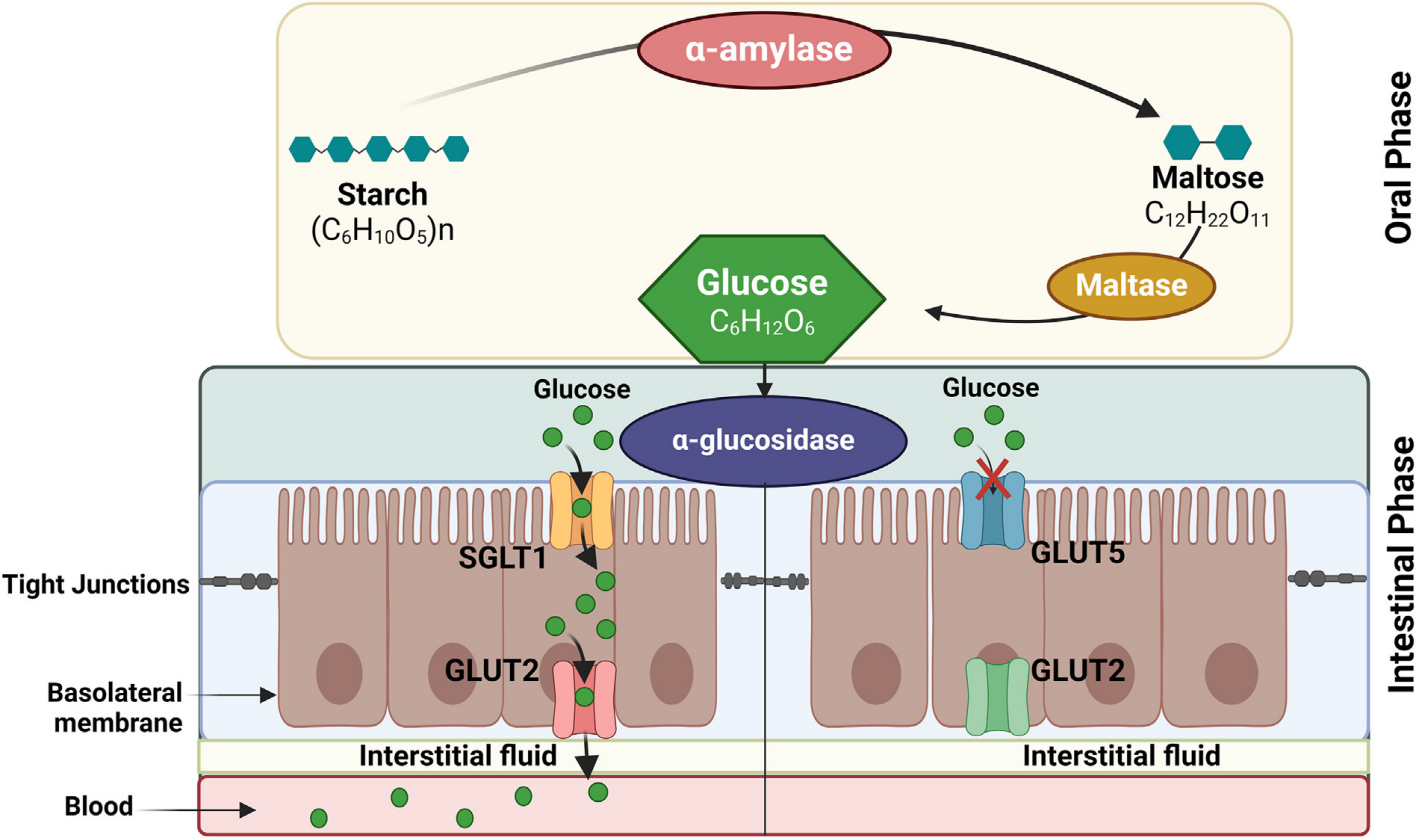
Systemic mechanisms of action of carbohydrate digesting enzymes and glucose transporters (GLUTs) in facilitating cellular glucose uptake across various phases of the human gastrointestinal/digestive tract.

After a meal is ingested, the initial stage of carbohydrate breakdown occurs in the mouth where salivary α-amylase hydrolyses α (1→4) bonds in large polysaccharides like starch and glycogen into disaccharides such as maltose [55,56]. Carbohydrates that remain undigested in the partially digested food bolus exiting the stomach [57] are further broken down into monosaccharides by the α-glucosidase enzyme located in the brush-border of enterocytes in the jejunum of the small intestine, where they are absorbed in the upper jejunum [58]. In this postprandial state, families of glucose transporters or carriers in the small intestine become activated, facilitating the transport of diet-derived monosaccharides (glucose) predominantly through SGLT1 and GLUT2 into the epithelial cells of the small intestine [59,60]. As a result, excessive glucose absorption during carbohydrate digestion in the digestive tract contributes to postprandial hyperglycemia or T2DM, as illustrated in Figure 3. Therefore, inhibiting the breakdown of carbohydrates into simple sugars, thereby reducing glucose absorption and/or transport across the small intestine, emerges as a strategic target for individuals experiencing elevated blood glucose concentrations after meals or postprandial hyperglycemia.

GLUTs, also known as membrane transporter proteins, facilitate the transport of glucose across the plasma membrane. GLUTs consist of ∼500 amino acid residues and are classified into various groups based on their amino acid sequences [61]. Among the well-studied GLUTs involved in intestinal glucose absorption and transportation are SGLT1, GLUT2, and GLUT5, which facilitate the transport of dietary fructose across the intestinal apical membrane in its monosaccharides form. Studies on glucose uptake have indicated that DPs interact directly with glucose transporters to modulate the rate of glucose absorption [[62], [63], [64]]. Glucose absorption in the intestine primarily occurs through active transport mediated by the sodium-dependent glucose transporter SGLT1 and facilitated sodium-independent transport mediated by the GLUT2 [65,66]. These studies have demonstrated that DPs can reduce intestinal glucose absorption and lower postprandial plasma glucose by inhibition of active uptake via SGLT1 and facilitated transport by GLUT2 [64]. Compounds such as chlorogenic, ferulic, caffeic, and tannic acids, quercetin monoglucosides [67], tea catechins [68], and naringenin [69] inhibit glucose transport by SGLT1, which is sodium dependent. Similarly, anthocyanin-rich berry extract inhibits glucose uptake in human intestinal Caco-2 cells, suggesting that berry flavonoids may regulate postprandial glycemia by reducing GLUT2 expression [70].

Under sodium-dependent conditions, glucose uptake can be inhibited by flavonoid glycosides and nonglycosylated polyphenols, while aglycones and phenolic acids show no effect. Conversely, under sodium-free conditions, aglycones and nonglycosylated polyphenols can inhibit glucose uptake, whereas glycosides and phenolic acids remain ineffective. This suggested that aglycones inhibit facilitated glucose uptake, whereas glycosides inhibit the active transport of glucose [71]. Barik et al. [72] tested a range of physiologically relevant concentrations (0.66–66 μg/mL) of anthocyanins and other phenolics from various berries on intestinal GLUTs *in vitro*. They discovered that anthocyanins were unable to cross the intestinal epithelium and did not inhibit GLUTs, whereas other phenolics at the same concentrations did inhibit them. The reason could be attributed to the following: *1*) low bioavailability of anthocyanins in the gastrointestinal tract; *2*) insufficient effective concentrations of anthocyanins to inhibit the GLUTs; or *3*) structural differences within the broad class of DPs.

Enzymatic hydrolysis of polyphenolic compounds derived from a variety of herbs, spices, and seeds significantly increased phenolic acids and flavonols. This increase subsequently reduced glucose transport across Caco-2 intestinal cells by interacting with the GLUT2 transporter family, leading to decreased glucose absorption and suggesting potential hypoglycemic effects [73]. These findings indicate that enzymatic hydrolysis of DPs may modulate glucose transporters, potentially contributing to a positive impact on reducing postprandial hyperglycemia. It is also suggested that DPs influence peripheral glucose uptake in both insulin-sensitive and non–insulin-sensitive tissues [74]. Green rooibos extract, a herbal tea containing the polyphenol aspalathin, was studied for its effects, demonstrating increased glucose uptake in conditions without insulin and inducing phosphorylation of AMPK in L6 myotubes. The extract also promotes phosphorylation of Akt, which facilitates GLUT4 translocation in L6 myotubes [75].

Treatment with phytochemicals such as eugenol and arecoline significantly increased the expressions of GLUT4 and PI3K genes, leading to enhanced 2-deoxyglucose uptake in L6 myotubes, when compared with the standard oral hypoglycemic drugs such as metformin and 2,4-thiazolidinedione [76]. Increased deoxyglucose uptake was observed in differentiated C2C12 muscle cells and 3T3-L1 adipocytes treated with extracts from *Vaccinium angustifolium* (blueberry). The stem, leaf, and fruit extracts of *V angustifolium* are also reported to reduce apoptosis (i.e. programmed cell death) by 20%–33% in PC12 cells exposed to high glucose concentrations for 96 h, demonstrating protective effects against glucose cytotoxicity and exhibiting properties similar to insulin and glitazone [77].

In a 15-week-old obese Zucker rat (an insulin-resistant model), the beneficial effect of apple polyphenol extract on insulin sensitivity in skeletal muscle cells was assessed using a meal tolerance test. The apple polyphenol extract, in synergy with insulin, significantly enhanced insulin sensitivity and increased the glucose infusion rate by 45%. The increase in glucose uptake was mediated by GLUT4 translocation in muscle cells, involving the phosphoinositide 3-kinase and peroxisome proliferator-activated receptor (PPAR) γ signaling pathways [64]. Similarly, cinnamon extracts rich in DPs have shown a potential to enhance insulin signaling and GLUT4 translocation in Swiss albino mouse embryo fibroblast line 3T3-L1 adipocytes [78] and to increase glucose uptake in insulin-resistant rats induced by a high fructose diet [79,80]. The DPs’ mediated regulation of glucose metabolism, through increased or decreased glucose uptake, may offer beneficial effects in the treatment or management of T2DM.

## DPs Activate AMPK

**FIGURE 4:**
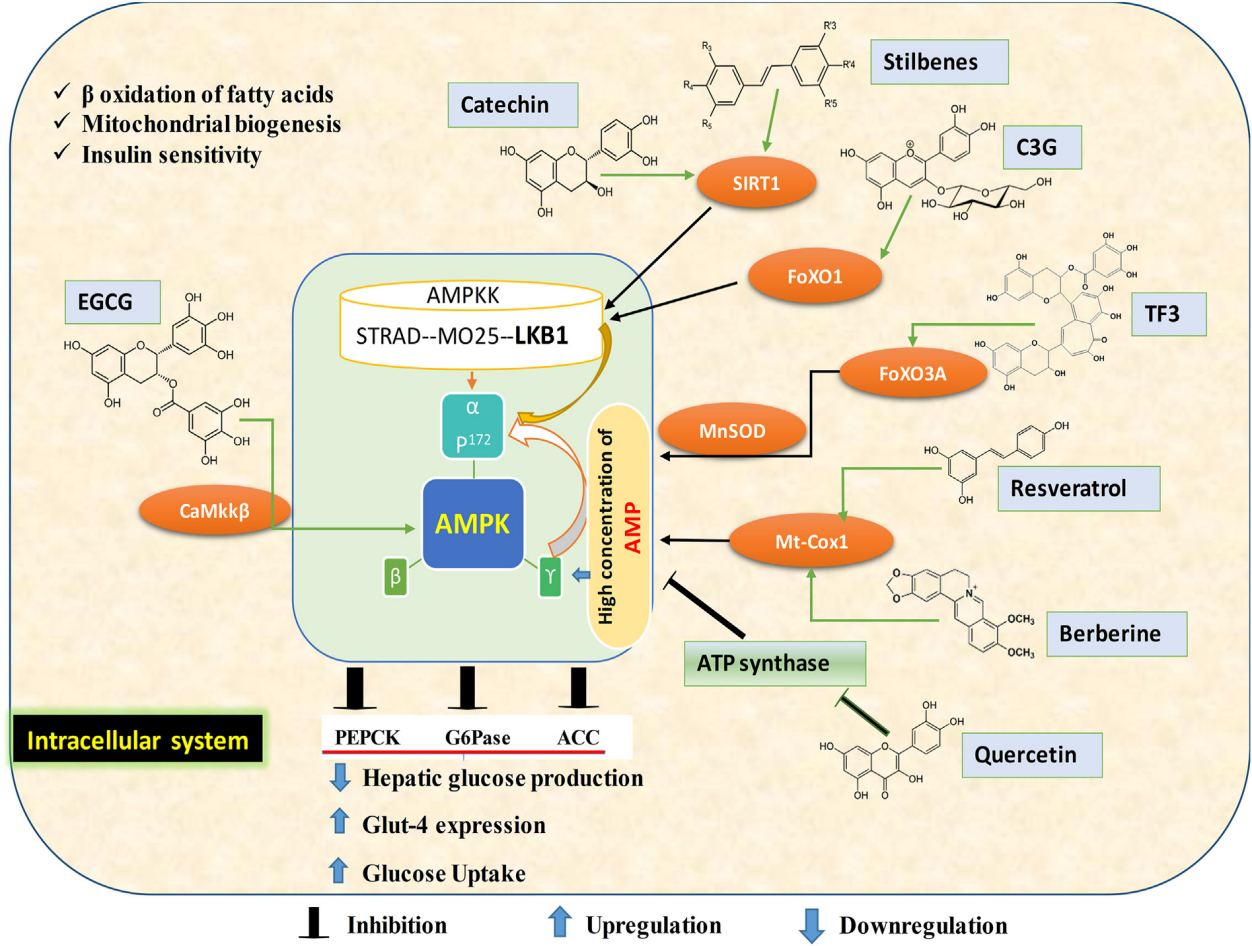
Dietary polyphenols (DPs) activate AMPK via associated pathways. The proposed mechanisms of action for individual DPs have been evidenced by various studies on the activation of AMP kinase (AMPK) within the intracellular system. Key DPs include epigallocatechin-3-gallate (EGCG), cyanidin-3-glucoside (C3G), theaflavin-3,3-digallate (TF3), liver kinase (LK) B1, acetyl-CoA carboxylase (ACC), Ca^2+^/calmodulin-dependent protein kinase (CaMkkβ), phosphoenolpyruvate carboxykinase (PEPCK), glucose-6-phosphate (G6pase) forkhead-boxO3A (FoXO3A), manganese superoxide dismutase (MnSOD), mitochondrial cytochrome C oxidase subunit-1 (Mt-Cox1), sirtuin (SIRT) 1, and cAMP.

AMPK is a key enzyme in cellular energy homeostasis and plays a major role in metabolic regulation. In hyperglycemic patients, activating AMPK can create a balance between interdependent elements, thereby maintaining blood glucose concentrations through subsequent physiologic processes. The activation of AMPK primarily occurs in response to changes in intracellular energy concentrations. High cellular energy requirements lead to increased concentrations of AMP. The increased AMP/ATP ratio activates upstream kinases, including the AMPKK (AMP-activated protein kinase kinase), liver kinase B1–STRAD–MO25 protein complex, which phosphorylates Thr172, resulting in AMPK activation. Another pathway for AMPK activation involves Ca^2+^/calmodulin-dependent protein kinase, which responds to an elevation of Ca^2+^ concentration in the cell cytoplasm. Activated AMPK reduces hepatic glucose production by inhibiting gluconeogenic enzymes phosphoenolpyruvate carboxykinase and glucose 6-phosphatase and facilitates the translocation of GLUT4 and GLUT1, leading to increased glucose uptake in adipocytes (Figure 4).

Activation of AMPK by DPs has been observed at much higher concentrations compared with that by the primary antidiabetic drug metformin [81]. A study on mice with T2DM indicated that anthocyanins activate the AMPK pathway, leading to an upregulation of GLUT4 transporter in skeletal muscle and a downregulation of gluconeogenesis in the liver in response to insulin [82]. In 3T3-L1 adipocytes, cyanidin-3-glucoside significantly enhanced the AMPK activation and protected high glucose-induced lipolysis [83].

Enhanced insulin-dependent glucose uptake through the activation of the AMPK pathway in in vitro conditions [84] shows potential for treating metabolic disorders like T2DM and obesity [85]. Similarly, Guo et al. [83] found that cyaniding-3-glucoside supplementation improved insulin sensitivity in high fat–fed and obese db/db mice via the transcription factor Forkhead box class O1. A recent study with ethanol extracts of *Alnus incana* and *Sarracenia purpurea* stimulated glucose uptake in C2C12 muscle cells by increasing GLUT4 expression and involving the AMPK pathway through Akt. The authors identified quercetin-3-O-galactoside and 3-O-α-L-arabinopyranoside as active antidiabetic phytochemicals in Canadian medicinal plants [86]. Eid et al. [87] concluded that the berries of *Vaccinium vitis-idaea* contain active quercetin and quercetin glycosides that enhance muscle cell glucose uptake in muscle cells. These active compounds exert their antidiabetic effects through the AMPK signaling pathway, specifically by the action of the quercetin aglycone on mitochondrial energy transduction.

Likewise, theaflavin-3,3′-gallate (TF3), a polyphenol found in black tea, activates AMPK and is essential for TF3 effects on PPARα upregulation, reversal of Forkhead box class O3A inactivation and insulin-induced suppression of manganese superoxide dismutase. The overexpression of manganese superoxide dismutase reduced intracellular lipid accumulation, indicating the activity of TF3 in 3T3-L1 adipocytes [88]. The antidiabetic action of stilbenes is believed to occur through the modulation of sirtuin 1 (a key metabolic sensor contributing to cellular regulation), which improved whole-body glucose homeostasis in insulin sensitivity in diabetic rats [89]. Chen et al. [90] reported that resveratrol likely prevents the onset of insulin resistance by inhibition of K^+^ ATP and K^+^ V channel in β cells in diabetic rats. However, when Hawley et al. [91] used isogenic cell lines expressing AMP-insensitive (R531G) AMPKγ2 subunit variants to investigate the mechanisms of AMPK activation by resveratrol, berberine, and quercetin, they found that these dietary compounds were unable to activate AMPK. Plausibly, these DPs could follow multiple pathways due to subtle structural differences within cells, and therefore, further studies on these specific DPs could elucidate the underlying molecular mechanisms in AMPK activation. Despite these discrepancies, none of the studies reported detrimental effects of these DPs, suggesting their potential in developing nutraceuticals warrants further investigation.

## Role of Incretins in Energy Homeostasis and Their Regulation by DPs on Dipeptidyl Peptidase IV Inhibition

Incretins are a group of metabolic gut hormones that help lower blood glucose concentrations by promoting insulin secretion from pancreatic β cells after a high-glucose meal. There are 2 types of incretins: GIP and GLP-1 [10]. GLP-1 is a neuropeptide secreted by enteroendocrine L-cells in the small and large intestines [92] and can help in reducing blood glucose plasma concentrations [93]. Similarly, GIP is a 42-amino acid peptide secreted by enteroendocrine K cells in the duodenum and jejunum and is present in the gastrointestinal mucosa. In T2DM conditions, GIP becomes less effective in glucose-dependent insulin secretion, and GLP-1 loses its insulinotropic properties due to its reduced ability to suppress glucagon function in the pancreas. Their reduced effectiveness in β-cell function can be problematic, making these compounds that activate GLP-1 receptors to enhance insulin secretion increasingly important.

Since individuals with T2DM often have reduced GLP-1 secretion, improving its bioactivity in an insulin-deficient state is challenging. A significant issue is the inhibiting of the dipeptidyl peptidase (DPP)-IV serine protease enzyme, which simultaneously cleaves GLP-1 after secretion following a meal, thus shortening its half-life. Therefore, GLP-1 secretagogues are promising targets in T2DM as they can enhance reduced incretin actions. While synthetic drugs like sitagliptin, vildagliptin, and saxagliptin are available to inhibit DPP-IV, emerging evidence suggests that natural DPs can also act as DPP-IV inhibitors, offering a diet-based therapeutic approach with minimal or no side effects. DPs have the potential to stimulate GLP-1 secretion from intestinal L-cells by increasing the half-life and inhibiting DPP-IV activity [94,95]. This results in increased insulin secretion through direct or indirect β-cell stimulation and improved insulin sensitivity in peripheral tissues, potentially mediated by the PPARγ transcription factor [95]. PPARγ activation leads to enhanced insulin secretion by upregulating the gene for incretin hormone GLP-1, along with other insulin growth factors, before the activation of adenylyl cyclase and cAMP when treated with a blend of berry polyphenols rich in anthocyanin, both directly in iNS-1E pancreatic β cells and following simulated absorption through human intestinal Caco-2 cells [96].

Primarily, the anthocyanin delphinidin-3-arabinoside has demonstrated the ability to modulate DPP-IV and its substrate GLP-1, leading to increased insulin secretion and upregulation of mRNA expression of insulin receptor–associated genes and proteins in pancreatic β cells [96]. The potential of *Hibiscus sabdariffa* Linn, known for its rich in anthocyanins content, was recently evaluated for its ability to induce GLP-1 secretion in the ileum and its effect on pancreatic β cells in diabetic rats. When they were fed with *H sabdariffa* (500 mg/kg of body weight), they had GLP-1 concentrations comparable with those of normal rats (*P* > 0.05), likely due to active ingredients such as leucosin, which binds to SGLT-1.

**FIGURE 5:**
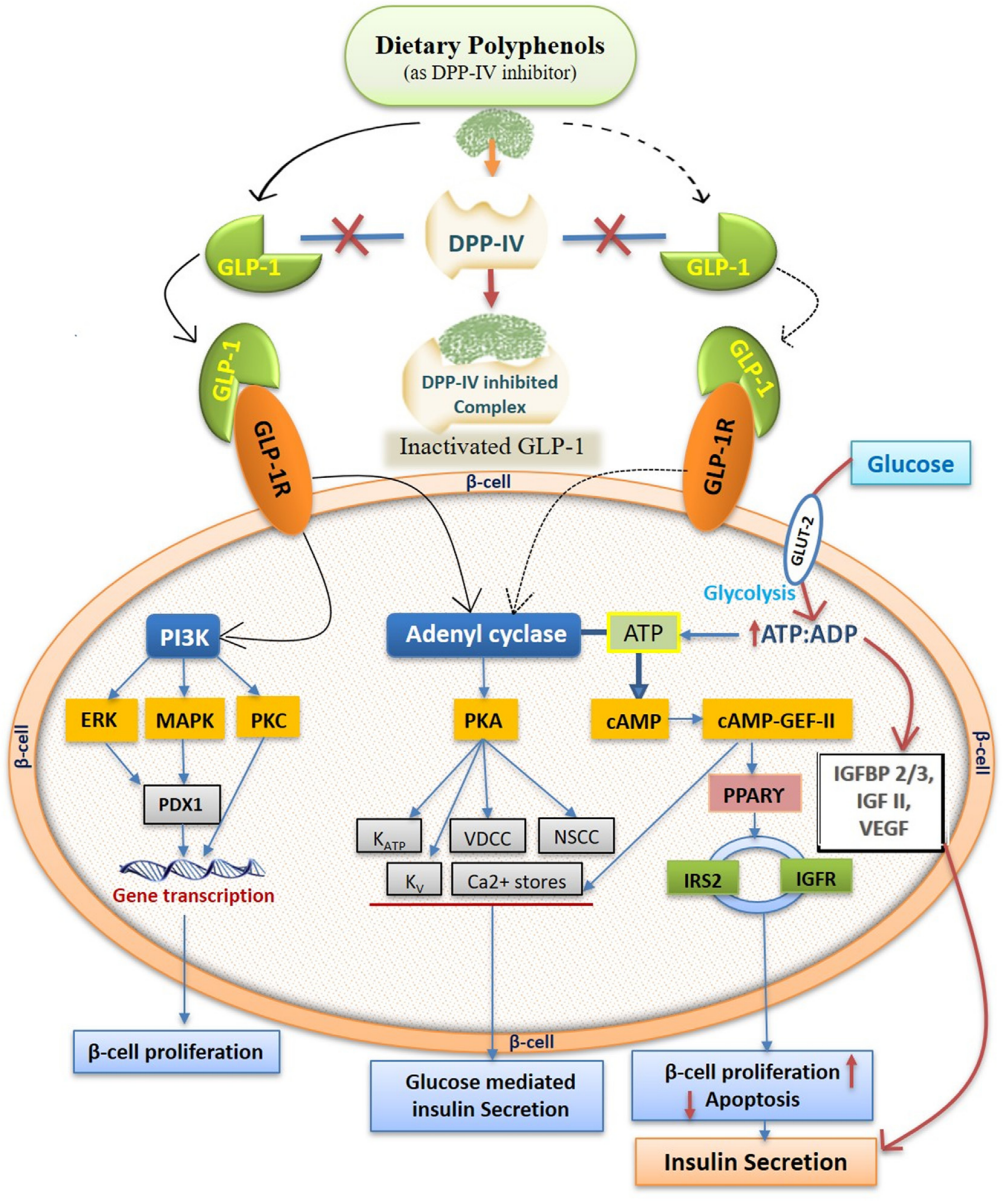
Schematic representation of dietary polyphenols (DPs) such as dipeptidyl peptidase (DPP)-IV inhibitor and their bioactivities on β cell for insulin secretion. The illustration shows the activation of GLP-1 receptors (GLP-1R) by DPs (illustrated by dotted arrows) and their proposed mechanism of action (indicated by solid arrows) for insulin secretion through various intracellular signaling responses. ERK, extracellular signal–regulated protein kinase; GEF, guanine nucleotide exchange factor; GLP, glucagon-like peptide; GLP-1R, glucagon-like peptide 1 receptor; IGFBP, insulin-like growth factor–binding protein; IGFR, insulin-like growth factor receptor; IRS, insulin receptor substrate; MAPK, mitogen-activated protein kinase; NSCC, nonselective cationic conductance; PDX1, pancreatic and duodenal homeobox 1—also known as insulin promotor factor 1; PI3K, phosphatidylinositol-4,5-bisphosphate 3-kinase; PK, protein kinase; VDCC, voltage-dependent calcium channel; VEGF, vascular endothelial growth factor.

Similarly, delphinidin from *H sabdariffa* binds to G protein–coupled receptors in the diabetes mellitus rat pancreas, which enhances GLP-1 secretion in the ileum [97]. These findings suggest that DPs contribute to insulin secretion in pancreatic β cells by promoting increased insulin release, enhancing β-cell proliferation, and reducing β-cell apoptosis. Furthermore, computational modeling of berry polyphenolics indicated that DPs (flavonoids) could directly dock into the active sites of DPP-IV through hydrogen bonding, thereby inhibiting its activity [98]. This suggests bioactive DPs may act as natural DPP-IV inhibitors, thereby enhancing GLP-1 incretins and functioning as agonists of GLP-1 receptors. GLP-1 stimulates pancreatic β-cell activation for glucose-induced insulin secretion through GLP-1 receptor activation. This process triggers the activation of adenylyl cyclase and cAMP via ATP, leading to the activation of secondary pathways such as the cAMP-dependent protein kinase and cAMP–guanine nucleotide exchange factor II [99]. A hypothetical illustration on how DPs activate GLP-1 receptors and inhibit DPP-IV is represented in Figure 5.

Ingesting coffee polyphenols enhanced the postprandial release of active GLP-1 amide in C57BL/6J mice, potentially due to increased insulin sensitivity through the cAMP-dependent pathway, which may have helped to reduce risk of developing T2DM [100]. Oral administration of coffee polyphenols increased diet-induced active GLP-1 secretion and decreased GIP release. Similarly, the addition of coffee polyphenols to human enteroendocrine NCI-H716 cells resulted in a dose-dependent increase in GLP-1 secretion [100].

In a 3-way, randomized crossover study, chlorogenic acid—a major polyphenol in coffee was found to reduce intestinal glucose absorption, affecting the GIP and GLP-1 profiles and suggesting an antagonistic effect on glucose transport in 9 healthy subjects [101]. Evidence supports the notion that coffee polyphenols, particularly chlorogenic acid, increase the secretion of incretins [102]. However, contrasting evidence from acute human studies indicated that ingesting 1 g of chlorogenic acid with 75 g of glucose did not significantly affect blood GLP-1 concentrations in overweight men [103]. Moreover, chlorogenic acid did not improve insulin sensitivity and had no significant effect on postprandial GLP-1 secretion in either NCI-H716 cells or rats [104]. These discrepancies raise questions about the efficacy of chlorogenic acid in aiding incretin secretion, necessitating further investigation into its mechanisms of action. It is important to note that bioactivities of individual DPs can vary across studies due to factors such as dosage, mode of treatment, and structural modification of the compound, which must be carefully considered to avoid inconsistencies in experimental settings.

## Conclusion and Future Prospects

This comprehensive review formed from various studies found that higher intake of DPs may decrease risk of developing T2DM, which is primarily due to the favorable effects of DPs on reducing blood glucose concentrations through several mechanism of actions (Table 1) [64,70,72,96,[104], [105], [106], [107], [108], [109], [110], [111], [112], [113], [114], [115], [116], [117], [118], [119], [120], [121], [122], [123], [124]]. Consuming DP-rich soft fruits with or shortly after a carbohydrate-rich meal can lower blood glucose concentrations by acting on carbohydrate metabolizing enzymes and glucose transporters. DPs were shown to enhance glucose uptake in both insulin-sensitive and non–insulin-sensitive tissues, influence intestinal glucose absorption and various signaling pathways, and improve secretion of intestinal GLP-1, extending its half-life and inhibiting DPP-IV. Epidemiologic studies also suggest that DPs can reduce postprandial hyperglycemia and help in preventing T2DM. However, results are inconsistent due to complex human physiology, and in some cases, the DPs may not have performed as expected, especially in the limited number of well-designed human trials conducted to date. Moreover, whole diets or drinks containing DPs make it difficult to identify the impact of individual dietary compounds on glucose homeostasis. Conversely, studies on specific DPs often miss the interaction between bioactive compounds and face challenges like the instability, availability, and cost-effectiveness of purified phenolic compounds.

**TABLE 1:** Bioactivities of DPs and their proposed antidiabetic mechanisms, supported by in vitro, animal, and human studies.

Bioactivities	Mechanism of actions	Reference
Anti-inflammatory	Reduces activation of proinflammatory markers such as IL-6 and TNF-α, while boosting the anti-inflammatory response	[105,106]
Antioxidant	Scavenge free radicals and chelators metal ions, which increases the hepatic superoxide dismutase (SOD) and catalase activities and thus activates the action of antioxidative enzymes	[[107], [108], [109]]
Protects pancreatic β cells against glucose toxicity	Protect β cells from damage by enhancing pancreatic insulin production and its release under high glucose concentrations	[[110], [111], [112]]
Increases insulin resistance	Achieves hypoglycemic effects by effectively facilitating glucose transporter (GLUT) 4–dependent glucose uptakes in cells and tissues	[64,113,114]
Inhibits α-amylases and α-glucosidases and regulates postprandial hyperglycemia	Lowes starch digestion in the mouth and its absorption in the small intestine by inhibiting the enzyme activities, thereby reducing glucose absorption and suppressing postprandial hyperglycemia	[72,[115], [116], [117]]
Reduced intestinal glucose absorption	Inhibits active uptake of intestinal glucose via sodium-dependent glucose transporter 1and facilitated transport through sodium-independent GLUT2	[70,72,118]
Reduced intestinal glucose transportation	Reduces the transport of carbohydrates from the small intestine into the portal vein and then to the liver, helping to prevent excessive glucose production	[119,120]
Activates AMP kinase	Increases AMP:ATP ratio by targeting direct or indirect molecular targets, thereby maintaining energy homeostasis	[121,122]
Incretin secretagogues	Stimulate L-cells to secrete glucagon-like peptide 1 and extends its half-life by inhibiting dipeptidyl peptidase-4, thereby enhancing insulin sensitivity	[96,104,123,124]

To develop safer and more effective nutraceuticals or functional foods, it is crucial to identify and characterize individual bioactive compounds from DP-rich foods. Notably, berries, rich in flavonoids, especially anthocyanins, have shown significant health benefits in regulating the underlying mechanisms of T2DM. Their availability and ease of consumption make berries promising nutraceuticals. However, current data on optimal doses and effective concentrations in humans and animals are inconsistent, requiring careful consideration before their industrial use. Furthermore, the above-discussed preclinical data show promising results and do not overstate the therapeutic potential of DPs, however, more clinical studies are needed to validate these findings and clarify where current evidence is still exploratory. Identifying the circulating metabolites and their systemic structural modifications is crucial in understanding the effects and bioactivities in human physiology. DPs’ bioactivities depend on various metabolites, not just their native forms, and considering T2DM complexity, it is essential to grasp how it eventually affects DPs’ pharmacology. Further research is needed to determine novel dietary compounds and advance the industrial use of DPs for managing T2DM.

## Author contributions

The authors’ responsibilities were as follows—SKB: conceived the study and wrote the original draft; SKB, SS, RA, SK, RC: performed investigation and formal analysis; SKB, RA, SK, RC: reviewed and edited the manuscript; SKB, JJK, RC: supervised the study; and all authors: have read and agreed to the published version of the manuscript.

## Funding

The authors reported no funding received for this study.

## Conflict of interest

The authors report no conflicts of interest.

## References

[1] WHO (2023).

[2] Keating S., El-Osta A. (2013). Epigenetic changes in diabetes. Clin. Genet..

[3] Sun H., Saeedi P., Karuranga S., Pinkepank M., Ogurtsova K., Duncan B.B. (2022). IDF Diabetes Atlas: global, regional and country-level diabetes prevalence estimates for 2021 and projections for 2045. Diabetes Res. Clin. Pract..

[4] GBD 2021 Diabetes Collaborators (2023). Global, regional, and national burden of diabetes from 1990 to 2021, with projections of prevalence to 2050: a systematic analysis for the Global Burden of Disease Study 2021. Lancet.

[5] International Diabetes Federation (2021).

[6] Jung U.J., Choi M.S. (2014). Obesity and its metabolic complications: the role of adipokines and the relationship between obesity, inflammation, insulin resistance, dyslipidemia and nonalcoholic fatty liver disease. Int. J. Mol. Sci..

[7] Ludwig D.D.S. (2002). The glycemic index—physiological mechanisms relating to obesity, diabetes, and cardiovascular disease. JAMA.

[8] Walker J.N., Ramracheya R., Zhang Q., Johnson P.R., Braun M., Rorsman P. (2011). Regulation of glucagon secretion by glucose: paracrine, intrinsic or both?. Diabetes Obes. Metab..

[9] Holst J.J., Vilsbøll T., Deacon C.F. (2009). The incretin system and its role in type 2 diabetes mellitus. Mol. Cell. Endocrinol..

[10] Dao T.M.A., Waget A., Klopp P., Serino M., Vachoux C., Pechere L. (2011). Resveratrol increases glucose induced GLP-1 secretion in mice: a mechanism which contributes to the glycemic control. PLoS One.

[11] Vilsboll T., Holst J.J. (2004). Incretins, insulin secretion and Type 2 diabetes mellitus. Diabetologia.

[12] Tsao R. (2010). Chemistry and biochemistry of dietary polyphenols. Nutrients.

[13] Han X., Shen T., Lou H. (2007). Dietary polyphenols and their biological significance. Int. J. Mol. Sci..

[14] Huang H.C., Szwerinski N.K., Nasrallah C., Huang Q., Chopra V., Venditti E.M. (2023). Lifestyle change program engagement in real-world clinical practice: a mixed-methods analysis. Transl. Behav. Med..

[15] Brunetti L., Kalabalik J. (2012). Management of type-2 diabetes mellitus in adults: focus on individualizing non-insulin therapies. Pharm. Ther..

[16] McCreight L.J., Bailey C.J., Pearson E.R. (2016). Metformin and the gastrointestinal tract. Diabetologia.

[17] Dujic T., Zhou K., Donnelly L.A., Tavendale R., Palmer C.N.A., Pearson E.R. (2015). Association of organic cation transporter 1 with intolerance to metformin in type 2 diabetes: a GoDARTS study. Diabetes.

[18] Ribon V., Johnson J.H., Camp H.S., Saltiel A.R. (1998). Thiazolidinediones and insulin resistance: peroxisome proliferator-activated receptor gamma activation stimulates expression of the CAP gene. Proc. Natl. Acad. Sci. U.S.A..

[19] Chiarelli F., Di Marzio D. (2008). Peroxisome proliferator-activated receptor-γ agonists and diabetes: current evidence and future perspectives. Vasc. Health Risk Manag..

[20] Cheng A.Y.Y., Fantus I.G. (2005). Oral antihyperglycemic therapy for type 2 diabetes mellitus. CMAJ.

[21] Hu F.B. (2011). Globalization of diabetes: the role of diet, lifestyle, and genes. Diabetes Care.

[22] Hsu W.C., Boyko E.J., Fujimoto W.Y., Kanaya A., Karmally W., Karter A. (2012). Pathophysiologic differences among Asians, Native Hawaiians, and Other Pacific Islanders and treatment implications. Diabetes Care.

[23] Carracher A.M., Marathe P.H., Close K.L. (2018). International Diabetes Federation 2017. J. Diabetes.

[24] WHO (2003). Diet, nutrition and the prevention of chronic diseases. World Health Organ. Tech. Rep. Ser..

[25] Chen L., Magliano D.J., Zimmet P.Z. (2011). The worldwide epidemiology of type 2 diabetes mellitus-present and future perspectives. Nat. Rev. Endocrinol..

[26] Galaviz K.I., Narayan K.M.V., Lobelo F., Weber M.B. (2018). Lifestyle and the prevention of type 2 diabetes: a status report, Am. J. Lifestyle. Med.

[27] Halliwell B. (2006). Polyphenols: antioxidant treats for healthy living or covert toxins?. J. Sci. Food Agric..

[28] Skibola C.F., Smith M.T. (2000). Potential health impacts of excessive flavonoid intake. Free Radic. Biol. Med..

[29] Scalbert A., Manach C., Morand C., Remesy C., Jimenez L. (2005). Dietary polyphenols and the prevention of diseases. Crit. Rev. Food Sci. Nutr..

[30] Torronen R., Sarkkinen E., Tapola N., Hautaniemi E., Kilpi K., Niskanen L. (2010). Berries modify the postprandial plasma glucose response to sucrose in healthy subjects. Br. J. Nutr..

[31] Govers C., Kasikci M.B., van der Sluis A.A., Mes J.J. (2018). Review of the health effects of berries and their phytochemicals on the digestive and immune systems. Nutr Rev.

[32] Gopalan A., Reuben S.C., Ahmed S., Darvesh A.S., Hohmann J., Bishayee A. (2012). The health benefits of blackcurrants. Food Funct.

[33] Barik S.K., Russell W.R., Dehury B., Cruickshank M., Moar K.M., Thapa D. (2019). Dietary phenolics other than anthocyanins inhibit PTP1B: an in vitro and in silico validation. Proc. Nutr. Soc..

[34] Barik S.K., Dehury B., Russell W.R., Moar K.M., Cruickshank M., Scobbie L. (2020). Analysis of polyphenolic metabolites from in vitro gastrointestinal digested soft fruit extracts identify malvidin-3-glucoside as an inhibitor of PTP1B. Biochem. Pharmacol..

[35] Pranprawit A., Heyes J.A., Molan A.L., Kruger M.C. (2015). Antioxidant activity and inhibitory potential of blueberry extracts against key enzymes relevant for hyperglycemia. J. Food Biochem..

[36] Castaneda-Ovando A., de Lourdes Pacheco-Hernandez M., Paez-Hernandez M.E., Rodriguez J.A., Galan-Vidal C.A. (2009). Chemical studies of anthocyanins: a review. Food Chem..

[37] Takikawa M., Inoue S., Horio F., Tsuda T. (2010). Dietary anthocyanin-rich bilberry extract ameliorates hyperglycemia and insulin sensitivity via activation of AMP-activated protein kinase in diabetic mice. J. Nutr..

[38] Belwal T., Nabavi S.F., Nabavi S.M., Habtemariam S. (2017). Dietary anthocyanins and insulin resistance: when food becomes a medicine. Nutrients.

[39] Jayaprakasam B., Vareed S.K., Olson L.K., Nair M.G. (2005). Insulin secretion by bioactive anthocyanins and anthocyanidins present in fruits. J. Agric. Food Chem..

[40] Lee S.G., Kim B., Yang Y., Pham T.X., Park Y.K., Manatou J.E. (2014). Berry anthocyanins suppress the expression and secretion of proinflammatory mediators in macrophages by inhibiting nuclear translocation of NF-kappa B independent of NRF2-mediated mechanism. J. Nutr. Biochem..

[41] Lee B., Lee M., Lefevre M., Kim H.R. (2014). Anthocyanins Inhibit Lipogenesis During Adipocyte Differentiation of 3T3-L1 Preadipocytes. Plant Foods Hum. Nutr..

[42] Tsuda T., Horio F., Uchida K., Aoki H., Osawa T. (2003). Dietary cyanidin 3-O-β-D-glucoside-rich purple corn color prevents obesity and ameliorates hyperglycemia in mice. J. Nutr..

[43] Collins B., Hoffman J., Martinez K., Grace M., Lila M.A., Cockrell C. (2016). A polyphenol-rich fraction obtained from table grapes decreases adiposity, insulin resistance and markers of inflammation and impacts gut microbiota in high-fat-fed mice. J. Nutr. Biochem..

[44] Guo X., Yang B., Tan J., Jiang J., Li D. (2016). Associations of dietary intakes of anthocyanins and berry fruits with risk of type 2 diabetes mellitus: a systematic review and meta-analysis of prospective cohort studies. Eur. J. Clin. Nutr..

[45] Guo H., Ling W. (2015). The update of anthocyanins on obesity and type 2 diabetes: experimental evidence and clinical perspectives. Rev. Endocr. Metab. Disord..

[46] He J., Giusti M.M. (2010). Anthocyanins: natural colorants with health-promoting properties. Annu. Rev. Food Sci. Technol..

[47] Cisowska A., Wojnicz D., Hendrich A.B. (2011). Anthocyanins as antimicrobial agents of natural plant origin. Nat. Prod. Commun..

[48] Matsumoto H., Ichiyanagi T., Iida H., Ito K., Tsuda T., Hirayama M. (2006). Ingested delphinidin-3-rutinoside is primarily excreted to urine as the intact form and to bile as the methylated form in rats. J. Agric. Food Chem..

[49] Matsumoto H., Inaba H., Kishi M., Tominaga S., Hirayama M., Tsuda T. (2001). Orally administered delphinidin 3-rutinoside and cyanidin 3-rutinoside are directly absorbed in rats and humans and appear in the blood as the intact forms. J. Agric. Food Chem..

[50] de Ferrars R.M., Czank C., Zhang Q., Botting N.P., Kroon P.A., Cassidy A. (2014). The pharmacokinetics of anthocyanins and their metabolites in humans. Br. J. Pharmacol..

[51] Mazza G., Kay C.D., Cottrell T., Holub B.J. (2002). Absorption of anthocyanins from blueberries and serum antioxidant status in human subjects. J. Agric. Food Chem..

[52] Mazza G., Miniati E. (1993).

[53] Kalt W., McDonald J.E., Ricker R.D., Lu X. (1999). Anthocyanin content and profile within and among blueberry species. Can. J. Plant Sci..

[54] Torronen R., Kolehmainen M., Sarkkinen E., Poutanen K., Mykkanen H., Niskanen L. (2013). Berries reduce postprandial insulin responses to wheat and rye breads in healthy women. J. Nutr..

[55] Williamson G. (2013). Possible effects of dietary polyphenols on sugar absorption and digestion. Mol. Nutr. Food Res..

[56] Hanhineva K., Torronen R., Bondia-Pons I., Pekkinen J., Kolehmainen M., Mykkanan H. (2010). Impact of dietary polyphenols on carbohydrate metabolism. Int. J. Mol. Sci..

[57] Liao D.H., Zhao J.B., Gregersen H. (2009). Gastrointestinal tract modelling in health and disease. World J. Gastroenterol..

[58] Puls W., Krause H.P., Muller L., Schutt H., Sitt R., Thomas G. (1984). Inhibitors of the rate of carbohydrate and lipid absorption by the intestine. Int. J. Obes..

[59] Kellett G.L., Brot-Laroche E., Mace O.J., Leturque A. (2008). Sugar absorption in the intestine: the role of GLUT2. Annu. Rev. Nutr..

[60] Satoh T., Igarashi M., Yamada S., Takahashi N., Watanabe K. (2015). Inhibitory effect of black tea and its combination with acarbose on small intestinal a-glucosidase activity. J. Ethnopharmacol..

[61] Mueckler M., Thorens B. (2013). The SLC2 (GLUT) family of membrane transporters. Mol. Aspects Med..

[62] Vishnu Prasad C.N., Suma Mohan S., Banerji A., Gopalakrishnapillai A. (2009). Kaempferitrin inhibits GLUT4 translocation and glucose uptake in 3T3-L1 adipocytes. Biochem. Biophys. Res. Commun..

[63] Vuong T., Martineau L.C., Ramassamy C., Matar C., Haddad P.S. (2007). Fermented Canadian lowbush blueberry juice stimulates glucose uptake and AMP-activated protein kinase in insulin-sensitive cultured muscle cells and adipocytes. Can. J. Physiol. Pharmacol..

[64] Manzano M., Giron M.D., Vilchez J.D., Sevillano N., El-Azem N., Rueda R. (2016). Apple polyphenol extract improves insulin sensitivity in vitro and in vivo in animal models of insulin resistance. Nutr. Metab (Lond)..

[65] Roeder P.V., Geillinger K.E., Zietek T.S., Thorens B., Koepsell H., Daniel H. (2014). The role of SGLT1 and GLUT2 in intestinal glucose transport and sensing. PLoS One.

[66] Kellett G.L., Brot-Laroche E. (2005). Apical GLUT2: a major pathway of intestinal sugar absorption. Diabetes.

[67] Cermak R., Landgraf S., Wolffram S. (2004). Quercetin glucosides inhibit glucose uptake into brush-border-membrane vesicles of porcine jejunum. Br. J. Nutr..

[68] Shimizu M., Kobayashi Y., Suzuki M., Satsu H., Miyamoto Y. (2000). Regulation of intestinal glucose transport by tea catechins. Biofactors.

[69] Li J.M., Che C.T., Lau C.B.S., Leung P.S., Cheng C.H.K. (2006). Inhibition of intestinal and renal Na+-glucose cotransporter by naringenin. Int. J. Biochem. Cell Biol..

[70] Alzaid F., Cheung H.M., Preedy V.R., Sharp P.A. (2013). Regulation of glucose transporter expression in human intestinal Caco-2 cells following exposure to an anthocyanin-rich berry extract. PLoS One.

[71] Johnston K., Sharp P., Clifford M., Morgan L. (2005). Dietary polyphenols decrease glucose uptake by human intestinal Caco-2 cells. FEBS Lett.

[72] Barik S.K., Russell W.R., Moar K.M., Cruickshank M., Scobbie L., Duncan G. (2020). The anthocyanins in black currants regulate postprandial hyperglycaemia primarily by inhibiting α-glucosidase while other phenolics modulate salivary α-amylase, glucose uptake and sugar transporters. J. Nutr. Biochem..

[73] Farrell T.L., Ellam S.L., Forrelli T., Williamson G. (2013). Attenuation of glucose transport across Caco-2 cell monolayers by a polyphenol-rich herbal extract: interactions with SGLT1 and GLUT2 transporters. Biofactors.

[74] Tsuda T. (2016). Recent progress in anti-obesity and anti-diabetes effect of berries. Antioxidants (Basel).

[75] Kamakura R., Son M.J., de Beer D., Joubert E., Miura Y., Yagasaki K. (2015). Antidiabetic effect of green rooibos (*Aspalathus linearis*) extract in cultured cells and type 2 diabetic model KK-A(y) mice. Cytotechnology.

[76] Prabhakar P.K., Doble M. (2011). Interaction of phytochemicals with hypoglycemic drugs on glucose uptake in L6 myotubes. Phytomedicine.

[77] Martineau L.C., Couture A., Spoor D., Benhaddou-Andaloussi A., Harris C., Meddah B. (2006). Anti-diabetic properties of the Canadian lowbush blueberry *Vaccinium angustifolium* Ait. Phytomedicine.

[78] Cao H., Polansky M.M., Anderson R.A. (2007). Cinnamon extract and polyphenols affect the expression of tristetraprolin, insulin receptor, and glucose transporter 4 in mouse 3T3-L1 adipocytes. Arch. Biochem. Biophys..

[79] Qin B., Nagasaki M., Ren M., Bajotto C., Oshida Y., Sato Y. (2004). Cinnamon extract prevents the insulin resistance induced by a high-fructose diet. Horm. Metab. Res..

[80] Qin B., Nagasaki M., Ren M., Bajotto G., Oshida Y., Sato Y. (2003). Cinnamon extract (traditional herb) potentiates in vivo insulin-regulated glucose utilization via enhancing insulin signaling in rats. Diabetes Res. Clin. Pract..

[81] Kim J., Yang G., Kim Y., Kim J., Ha J. (2016). AMPK activators: mechanisms of action and physiological activities. Exp. Mol. Med..

[82] Kurimoto Y., Shibayama Y., Inoue S., Soga M., Takikawa M., Ito C. (2013). Black soybean seed coat extract ameliorates hyperglycemia and insulin sensitivity via the activation of AMP-activated protein kinase in diabetic mice. J. Agric. Food Chem..

[83] Guo H., Xia M., Zou T., Ling W., Zhong R., Zhang W. (2012). Cyanidin 3-glucoside attenuates obesity-associated insulin resistance and hepatic steatosis in high-fat diet-fed and db/db mice via the transcription factor FoxO1. J. Nutr. Biochem..

[84] Park C.E., Kim M.J., Lee J.H., Min B.I., Bae H., Choe W. (2007). Resveratrol stimulates glucose transport in C2C12 myotubes by activating AMP-activated protein kinase. Exp. Mol. Med..

[85] Towler M.C., Hardie D.G. (2007). AMP-activated protein kinase in metabolic control and insulin signalling. Circ. Res..

[86] Shang N., Saleem A., Musallam L., Walshe-Roussel B., Badawi A., Cuerrier A. (2015). Novel approach to identify potential bioactive plant metabolites: pharmacological and metabolomics analyses of ethanol and hot water extracts of several Canadian medicinal plants of the Cree of Eeyou Istchee. PLoS One.

[87] Eid H.M., Martineau L.C., Saleem A., Muhammad A., Vallerand D., Benhaddou-Andaloussi A. (2010). Stimulation of AMP-activated protein kinase and enhancement of basal glucose uptake in muscle cells by quercetin and quercetin glycosides, active principles of the antidiabetic medicinal plant *Vaccinium vitis-idaea*. Mol. Nutr. Food Res..

[88] Ko H.J., Lo C.Y., Wang B.J., Chiou R.Y.Y., Lin S.M. (2015). Theaflavin-3,3’-digallate, a black tea polyphenol, stimulates lipolysis associated with the induction of mitochondrial uncoupling proteins and AMPK-FoxO3A-MnSOD pathway in 3T3-L1 adipocytes. J. Funct Foods..

[89] Milne J.C., Lambert P.D., Schenk S., Carney D.P., Smith J.J., Gagne D.J. (2007). Small molecule activators of SIRT1 as therapeutics for the treatment of type 2 diabetes. Nature.

[90] Chen W.P., Chi T.C., Chuang L.M., Su M.J. (2007). Resveratrol enhances insulin secretion by blocking K-ATP and K-V channels of beta cells. Eur. J. Pharmacol..

[91] Hawley S.A., Ross F.A., Chevtzoff C., Green K.A., Evans A., Fogarty S. (2010). Use of cells expressing γ subunit variants to identify diverse mechanisms of AMPK activation. Cell Metab..

[92] Kreymann B., Ghatei M.A., Williams G., Bloom S.R. (1987). Glucagon-like peptide-1 7-36: a physiological incretin in man. Lancet.

[93] Näslund E., Bogefors J., Skogar S., Grybäck P., Jacobsson H., Holst J.J. (1999). GLP-1 slows solid gastric emptying and inhibits insulin, glucagon, and PYY release in humans. Am. J. Physiol..

[94] Hlebowicz J., Hlebowicz A., Lindstedt S., Bjoergell O., Hoeglund P., Holst J.J. (2009). Effects of 1 and 3 g cinnamon on gastric emptying, satiety, and postprandial blood glucose, insulin, glucose-dependent insulinotropic polypeptide, glucagon-like peptide 1, and ghrelin concentrations in healthy subjects. Am. J. Clin. Nutr..

[95] Domínguez Avila J.A., Rodrigo García J., González Aguilar G.A., De La Rosa L.A. (2017). The antidiabetic mechanisms of polyphenols related to increased glucagon-like peptide-1 (GLP1) and insulin signalling. Molecules.

[96] Johnson M.H., de Mejia E.G. (2016). Phenolic compounds from fermented berry beverages modulated gene and protein expression to increase insulin secretion from pancreatic beta-cells in vitro. J. Agric. Food Chem..

[97] Kartinah N.T., Fadilah F., Ibrahim E.I., Suryati Y. (2019). The potential of *Hibiscus sabdariffa* Linn in inducing glucagon-like peptide-1 via SGLT-1 and GLPR in DM rats. Biomed. Res. Int..

[98] Fan J., Johnson M.H., Lila M.A., Yousef G., de Mejia E.G. (2013). Berry and citrus phenolic compounds inhibit dipeptidyl peptidase IV: implications in diabetes management. Evid. Based Complement. Alternat. Med..

[99] MacDonald P.E., El-kholy W., Riedel M.J., Salapatek A.M.F., Light P.E., Wheeler M.B. (2002). The multiple actions of GLP-1 on the process of glucose-stimulated insulin secretion. Diabetes.

[100] Fujii Y., Osaki N., Hase T., Shimotoyodome A. (2015). Ingestion of coffee polyphenols increases postprandial release of the active glucagon-like peptide-1 (GLP-1(7-36)) amide in C57BL/6J mice. J. Nutr. Sci..

[101] Johnston K.L., Clifford M.N., Morgan L.M. (2003). Coffee acutely modifies gastrointestinal hormone secretion and glucose tolerance in humans: glycemic effects of chlorogenic acid and caffeine. Am. J. Clin. Nutr..

[102] McCarty M.F. (2005). A chlorogenic acid-induced increase in GLP-1 production may mediate the impact of heavy coffee consumption on diabetes risk. Med. Hypotheses..

[103] Olthof M.R., Van Dijk A.E., Deacon C.F., Heine R.J., Van Dam R.M. (2011). Acute effects of decaffeinated coffee and the major coffee components chlorogenic acid and trigonelline on incretin hormones. Nutr. Metab (Lond)..

[104] Tunnicliffe J.M., Eller L.K., Reimer R.A., Hittel D.S., Shearer J. (2011). Chlorogenic acid differentially affects postprandial glucose and glucose-dependent insulinotropic polypeptide response in rats. Appl. Physiol. Nutr. Metab..

[105] Wu S., Yano S., Chen J., Hisanaga A., Sakao K., He X. (2017). Polyphenols from *Lonicera caerulea* L. berry inhibit LPS-induced inflammation through dual modulation of inflammatory and antioxidant mediators. J. Agric. Food Chem..

[106] Carito V., Ciafrè S., Tarani L., Ceccanti M., Natella F., Iannitelli A. (2015). TNF-α and IL-10 modulation induced by polyphenols extracted by olive pomace in a mouse model of paw inflammation. Ann. Ist. Super Sanita..

[107] Pandey K.B., Rizvi S.I. (2009). Plant polyphenols as dietary antioxidants in human health and disease. Oxid. Med. Cell Longev..

[108] Dembinska-Kiec A., Mykkänen O., Kiec-Wilk B., Mykkänen H. (2008). Antioxidant phytochemicals against type 2 diabetes. Br. J. Nutr..

[109] Seeram N.P. (2008). Berry fruits: compositional elements, biochemical activities, and the impact of their intake on human health, performance, and disease. J. Agric. Food Chem..

[110] Dall’Asta M., Bayle M., Neasta J., Scazzina F., Bruni R., Cros G. (2015). Protection of pancreatic beta-cell function by dietary polyphenols, Phytochem. Rev..

[111] Lee J.S., Kim Y.R., Song I.G., Ha S.J., Kim Y.E., Baek N.I. (2015). Cyanidin-3-glucoside isolated from mulberry fruit protects pancreatic beta-cells against oxidative stress-induced apoptosis. Int. J. Mol. Med..

[112] Zhang B., Kang M., Xie Q., Xu B., Sun C., Chen K. (2011). Anthocyanins from Chinese bayberry extract protect beta cells from oxidative stress-mediated injury via HO-1 upregulation. J. Agric. Food Chem..

[113] Yamashita Y., Wang L., Nanba F., Ito C., Toda T., Ashida H. (2016). Procyanidin promotes translocation of glucose transporter 4 in muscle of mice through activation of insulin and AMPK signaling pathways. PLoS One.

[114] Russo B., Picconi F., Malandrucco I., Frontoni S. (2019). Flavonoids and insulin-resistance: from molecular evidences to clinical trials. Int. J. Mol. Sci..

[115] Grussu D., Stewart D., McDougall G.J. (2011). Berry polyphenols inhibit alpha-amylase in vitro: identifying active components in rowanberry and raspberry. J. Agric. Food Chem..

[116] McDougall G.J., Shpiro F., Dobson P., Smith P., Blake A., Stewart D. (2005). Different polyphenolic components of soft fruits inhibit a-amylase and a-glycosidase. J. Agric. Food Chem..

[117] Castro-Acosta M.L., Stone S.G., Mok J.E., Mhajan R.K., Fu C.I., Lenihan-Geels G.N. (2017). Apple and blackcurrant polyphenol-rich drinks decrease postprandial glucose, insulin and incretin response to a high-carbohydrate meal in healthy men and women. J. Nutr. Biochem..

[118] Manzano S., Williamson G. (2010). Polyphenols and phenolic acids from strawberry and apple decrease glucose uptake and transport by human intestinal Caco-2 cells. Mol. Nutr. Food Res..

[119] Moore M.C., Coate K.C., Winnick J.J., An Z., Cherrington A.D. (2012). Regulation of hepatic glucose uptake and storage in vivo. Adv. Nutr..

[120] Redan B.W., Buhman K.K., Novotny J.A., Ferruzzi M.G. (2016). Altered transport and metabolism of phenolic compounds in obesity and diabetes: implications for functional food development and assessment. Adv. Nutr..

[121] Zang M., Xu S., Maitland-Toolan K.A., Zuccollo A., Hou X., Jiang B. (2006). Polyphenols stimulate AMP-activated protein kinase, lower lipids, and inhibit accelerated atherosclerosis in diabetic LDL receptor-deficient mice. Diabetes.

[122] Tang X., Shen T., Jiang X., Xia M., Sun X., Guo H. (2015). Purified anthocyanins from bilberry and black currant attenuate hepatic mitochondrial dysfunction and steatohepatitis in mice with methionine and choline deficiency. J. Agric. Food Chem..

[123] Bozzetto L., Annuzzi G., Pacini G., Costabile G., Vetrani C., Vitale M. (2015). Polyphenol-rich diets improve glucose metabolism in people at high cardiometabolic risk: a controlled randomised intervention trial. Diabetologia.

[124] Hoggard N., Cruickshank M., Moar K.M., Bestwick C., Holst J.J., Russell W. (2013). A single supplement of a standardised bilberry (*Vaccinium myrtillus* L.) extract (36 % wet weight anthocyanins) modifies glycaemic response in individuals with type 2 diabetes controlled by diet and lifestyle. J. Nutr. Sci..

